# *Sultr4;1 *mutant seeds of Arabidopsis have an enhanced sulphate content and modified proteome suggesting metabolic adaptations to altered sulphate compartmentalization

**DOI:** 10.1186/1471-2229-10-78

**Published:** 2010-04-28

**Authors:** Hélène Zuber, Jean-Claude Davidian, Markus Wirtz, Rüdiger Hell, Maya Belghazi, Richard Thompson, Karine Gallardo

**Affiliations:** 1 UMR102 Genetics and Ecophysiology of Grain legumes, INRA, F-21000 Dijon, France; 2 UMR5004 Biochemistry and Plant Molecular Physiology, Montpellier SupAgro/CNRS/INRA/Université MontpellierII, F-34060 Montpellier, France; 3 Heidelberg Institute of Plant Sciences, University of Heidelberg, D-69120 Heidelberg, Germany; 4 Proteomic Analysis Center of Marseille, IFR Jean Roche, F-13916 Marseille Cedex 20, France

## Abstract

**Background:**

Sulphur is an essential macronutrient needed for the synthesis of many cellular components. Sulphur containing amino acids and stress response-related compounds, such as glutathione, are derived from reduction of root-absorbed sulphate. Sulphate distribution in cell compartments necessitates specific transport systems. The low-affinity sulphate transporters SULTR4;1 and SULTR4;2 have been localized to the vacuolar membrane, where they may facilitate sulphate efflux from the vacuole.

**Results:**

In the present study, we demonstrated that the *Sultr4;1 *gene is expressed in developing Arabidopsis seeds to a level over 10-fold higher than the *Sultr4;2 *gene. A characterization of dry mature seeds from a *Sultr4;1 *T-DNA mutant revealed a higher sulphate content, implying a function for this transporter in developing seeds. A fine dissection of the *Sultr4;1 *seed proteome identified 29 spots whose abundance varied compared to wild-type. Specific metabolic features characteristic of an adaptive response were revealed, such as an up-accumulation of various proteins involved in sugar metabolism and in detoxification processes.

**Conclusions:**

This study revealed a role for SULTR4;1 in determining sulphate content of mature Arabidopsis seeds. Moreover, the adaptive response of *sultr4;1 *mutant seeds as revealed by proteomics suggests a function of SULTR4;1 in redox homeostasis, a mechanism that has to be tightly controlled during development of orthodox seeds.

## Background

In recent decades, sulphur deficiency has become an increasing problem in crops of many countries, notably in Western Europe, leading to sulphur deficiency symptoms and resulting in decreased crop yields and quality parameters [1-3]. In this context, sulphur acquisition and metabolism in plants has become a major concern for research and crop improvement. Sulphur is an essential macronutrient required for plant growth. It is mainly acquired by the plant roots as inorganic sulphate and then distributed within the tissues [4]. After entering the cells, sulphate is reduced to sulphide with any excess sulphate being stored in the vacuole [5-7]. Sulphate assimilation leads to the synthesis of many compounds, including sulphur amino acids (cysteine and methionine), secondary products (glucosinolates and flavonoids) and numerous essential metabolites derived from sulphur-containing amino acids, such as cofactors (e.g. glutathione, *S*-adenosylmethionine, coenzyme A) and vitamins (e.g. biotin and thiamine). Therefore, sulphur assimilation is an essential part of plant metabolism and plays a crucial role in many plant processes. For example, as part of glutathione, sulphur has a role in the maintenance of the cellular redox balance and mitigation of oxidative stress in response to environmental changes. Furthermore, as constituent of sulphur amino acids, sulphur is tightly connected to protein synthesis and metabolism and is of great importance in terms of nutritional value. Indeed, animals and humans are unable to synthesize methionine and are dependent on dietary sources of this amino acid. Seeds of many agronomically important crops, including grain legumes, while rich in proteins, are deficient in the sulphur amino acids. Tabe and Droux [8] demonstrated that sulphate is the dominant form of sulphur found in the phloem supplying pods during lupin seed development and that the seed is able to reduce and assimilate sulphate in sufficient quantities for its needs. Therefore, identifying the mechanisms involved in sulphate acquisition and distribution in the developing seed is relevant to seed quality improvement.

The movement of sulphate around the plant and between cell compartments is facilitated by specific sulphate transporters (SULTR), which are encoded by a large gene family, consisting of 14 members in Arabidopsis and rice. Phylogenetic analyses indicate this gene family can be divided into four closely related groups (SULTR1 to 4) each with 12 membrane-spanning domains and a STAS (Sulphate Transporter and Anti-sigma Antagonist) domain at the carboxy-terminus [9], and a fifth more distinct group, SULTR5, lacking the STAS domain [4]. Notably, the Arabidopsis *Sultr5;2 *gene has recently been demonstrated to encode a high-affinity root molybdate transporter [10], which raises the question of the role of group 5 genes in sulphate transport. Considering differences in their kinetics and expression patterns, the different groups have been proposed to represent functional subtypes [11,12]. Group 1 and 2 sulphate transporters, which are localised at the plasma membrane, have been the subject of several studies and are the best characterized groups. Members of group 1 represent high-affinity transporters that facilitate uptake of sulphate by the root (SULTR1;1 and SULTR1;2) or translocation of sulphate from source-to-sink organs (SULTR1;3) [13-17]. Group 2 is composed of low-affinity sulphate transporters whose gene products may rather play a role in vascular tissues, faciliting the translocation of sulphate around the plant [[4,14] and [18]]. Group 3 is composed of low affinity transporters localized at the plasma membrane. The data available in the litterature indicate a differential expression of these genes in plant tissues, which is not stimulated by sulphur deficiency [19], and a role for SULTR3;5 in the root-to-shoot transport of sulphate in cooperation with SULTR2;1 in Arabidopsis [20].

Unlike groups 1 to 3, group 4 sulphate transporters have been localized to the vacuolar membrane: a SULTR4;1-GFP fusion protein was specifically accumulated in the vacuoles of roots and hypocotyls from young seedlings [21]. The *Sultr4;1 *gene was shown to be expressed in roots under sulphur-sufficient and deficient conditions, where it may play a role in the efflux of sulphate from the vacuolar lumen into the cytoplasm and influence the vacuolar storage capacity for sulphate [21]. In contrast, *Sultr4;2 *gene expression was shown to be highly inducible by sulphur limitation in the same tissue. The sultr4;1/sultr4;2 double knock-out mutants contained higher amounts of sulphate than did wild-type plants. Comparison of single and *sultr4;1*/*sultr4;2 *double knock-out mutants suggested that *Sultr4;1 *plays a major role and *Sultr4;2 *has a supplementary function [21]. Although sulphate transport has been extensively studied in roots, to date there has been no investigation of the roles of individual sulphate transporters within seeds. The vacuole may certainly play a role in the storage and unloading of sulphate within the developing seed, in which case SULTR4 transporters would be key players.

In this study, we demonstrate that, the Arabidopsis *Sultr4;1 *gene is strongly expressed in developing seeds and that its disruption significantly increases seed sulphate content, suggesting that SULTR4;1 is involved in the efflux of sulphate from vacuoles within developing seeds. Furthermore, a proteome analysis of *Sultr4;1 *mutant seeds reveals metabolic modulations suggesting adaptations to altered sulphate conpartmentation, which implicates SULTR4;1-mediated sulphate transport in establishment of defence mechanisms against oxidative stress during seed development.

## Results

### *Sultr4;1 *is strongly expressed during seed filling

**Figure 1:**
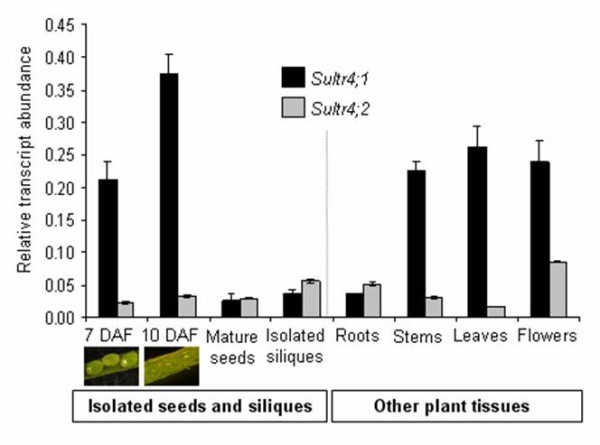
**Expression profiles of genes encoding SULTR4;1 and SULTR4;2 in plant tissues**. The relative mRNA abundance was estimated by qRT-PCR in developing Arabidopsis seeds removed from siliques, in mature seeds, in siliques collected at 7-10 day after flowering DAF), and in other tissues of plants grown in sulphur-sufficient conditions. Developing seeds were collected during embryogenesis (7 days after flowering), and early seed filling (10 day after flowering). Bars represent the mean ± SE of three independent measurements, which are representative of two independent biological experiments.

We first analysed by Real-Time Quantitative Reverse Transcription-PCR (qRT-PCR) gene expression levels of the Arabidopsis sulphate transporters belonging to group 4 (SULTR4;1 and SULTR4;2) (Figure 1). The analysis was performed on isolated seeds [7 and 10 days after flowering (DAF) corresponding to embryogenesis and seed filling stages, respectively], on isolated siliques (at 7-10 DAF) and for other tissues (leaves, flowers, roots, and stems), all collected during the reproductive growth phase of plants grown under sulphur-sufficient conditions. In addition, we compared qRT-PCR expression profiles by using an available microarray-based transcriptomics dataset ([22], see Additional file 1). The qRT-PCR data were consistent with the microarray dataset, but as commonly observed, the dynamic range of expression was higher with qRT-PCR.

The combined set of expression data (see Figure 1 and Additional file 1) showed an expression of both genes in various plant tissues during reproductive growth, but with different levels and patterning of expression. First, *Sultr4;1 *was strongly expressed in developing seeds, whereas *Sultr4;2 *was preferentially expressed in flowers. Second, microarray expression data available for roots of 10-day-old plants subjected to sulphur starvation for 24 h revealed that the two genes respond differently to a limitation in sulphur supply, *Sultr4;1 *and *Sultr4;2 *expression being up-regulated by 1.7 and 3.7 fold, respectively, in these conditions (see Additional file 1). Third, the two genes were differentially expressed during seed development: *Sultr4;1 *was highly expressed at the onset of seed filling, whereas *Sultr4;2 *was expressed at almost constitutive levels from embryogenesis to the dry mature stage. Finally, the qRT-PCR data revealed that the *Sultr4;1 *gene was more highly expressed than *Sultr4;2 *in most plant organs (Figure 1). Notably, *Sultr4;1 *was up to 10-fold more expressed in the isolated developing seeds at 7 and 10 DAF as compared with *Sultr4;2*, whereas both transcripts were similarly abundant in the isolated siliques at the same stages. These data suggested the SULTR4;1 may have an important function within the developing seed, which had not previously been reported and merited further investigations.

### Sulphate content is elevated in *SULTR4;1 *mature seeds

To investigate the role of SULTR4;1 in determining seed sulphate content, we used a s*ultr4;1 *T-DNA insertion line from the Arabidopsis SALK collection [23]. The T-DNA was located in the STAS domain http://atensembl.arabidopsis.info/index.html critical for both the activity and biosynthesis/stability of sulphate transporters [24]. The presence of the insertion in plants was confirmed by PCR (data not shown). Mature seeds were collected from homozygous mutant plants and from the corresponding wild-type (Columbia, Col-O). *Sultr4;1 *gene expression was measured by qRT-PCR in mature seeds of wild-type and *sultr4;1 *mutant plants, from three biological replicates. In the mutant line, the accumulation of the corresponding intact mRNA decreased strongly (94-99% decrease, see Additional file 2), suggesting that the T-DNA insertion altered dramatically transcription and/or mRNA stability.

**Figure 2:**
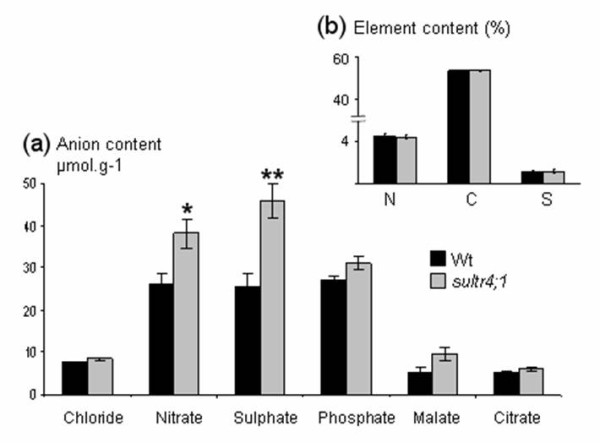
**Analysis of anion, total nitrogen, carbon and sulphur contents in mature Arabidopsis seeds of wild type (Wt) and *sultr4;1 *mutant plants**. a) Seed anion content (μmol.g-1) determined by high performance ionic chromatography. b) Seed nitrogen (N), carbon (C) and sulphur (S) contents (%). Results are representative of three biological experiments. Bars represent the mean ± SE of six measurements (at least two technical replicates from each biological replicate). * and ** indicate p < 0.05 and p < 0.01, respectively.

**Figure 3:**
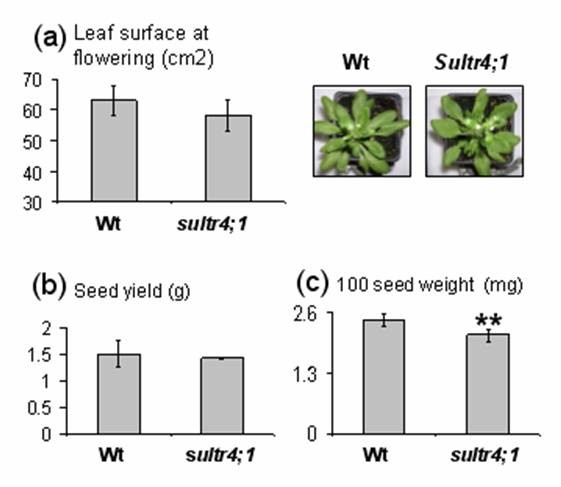
**Projected rosette leaf surface at flowering a), seed yield b), and seed weight c) of wild-type and *sultr4;1 *mutant plants**. Each bar represents the mean ± SE of at least three biological replicates. The data were submitted to variance analysis. * and ** indicate p < 0.05 and p < 0.01, respectively.

The profiles and contents of major inorganic and organic anions (sulphate, nitrate, phosphate, chloride, malate and citrate) and the overall sulphur content were determined (Figure 2). Interestingly, in mature s*ultr4;1 *seeds, the average sulphate content was about 1.7 fold higher than that of wild-type (Figure 2a), whereas seed sulphur content was unchanged (Figure 2b). The sulphate form represents a significant fraction of sulphur in mature seeds: 7.7% and 13.24% of total sulphur content respectively in wild-type and *sultr4;1 *mutant seeds. With respect to the s*ultr4;1 *plant phenotype, no significant difference in seed yield, leaf surface, or onset of flowering was observed (Figure 3). Only a slight decrease in s*ultr4;1 *seed weight was observed compared to wild-type (Figure 3c). These data suggest sulphur flux into developing seeds may not be affected in *sultr4;1 *mutant and the increase in seed sulphate content is therefore not related to a drastic perturbation of s*ultr4;1 *vegetative growth. This was further confirmed by estimating the level of sulphate in the seed compartment (sulphate content × seed mass per plant) and per seed (sulphate content × 1-seed weight), which increased by ~60% in s*ultr4;1 *seeds compared to wild-type (see Additional file 3).

For the other anions measured, only nitrate content was found to increase in s*ultr4;1 *seeds but nitrate levels in the seed compartment or per seed were not significantly different compared to wild-type (see Additional file 3), suggesting s*ultr4;1 *is particularly affected in seed sulphate content. The significant increased pool of sulphate in mature seeds of the s*ultr4;1 *mutant could be related to a reduced efflux of sulphate from the vacuoles during seed development, therefore no longer accessible for its assimilatory reduction.

### Alterations in protein quantities in *sultr4;1 *mature seeds

**Figure 4:**
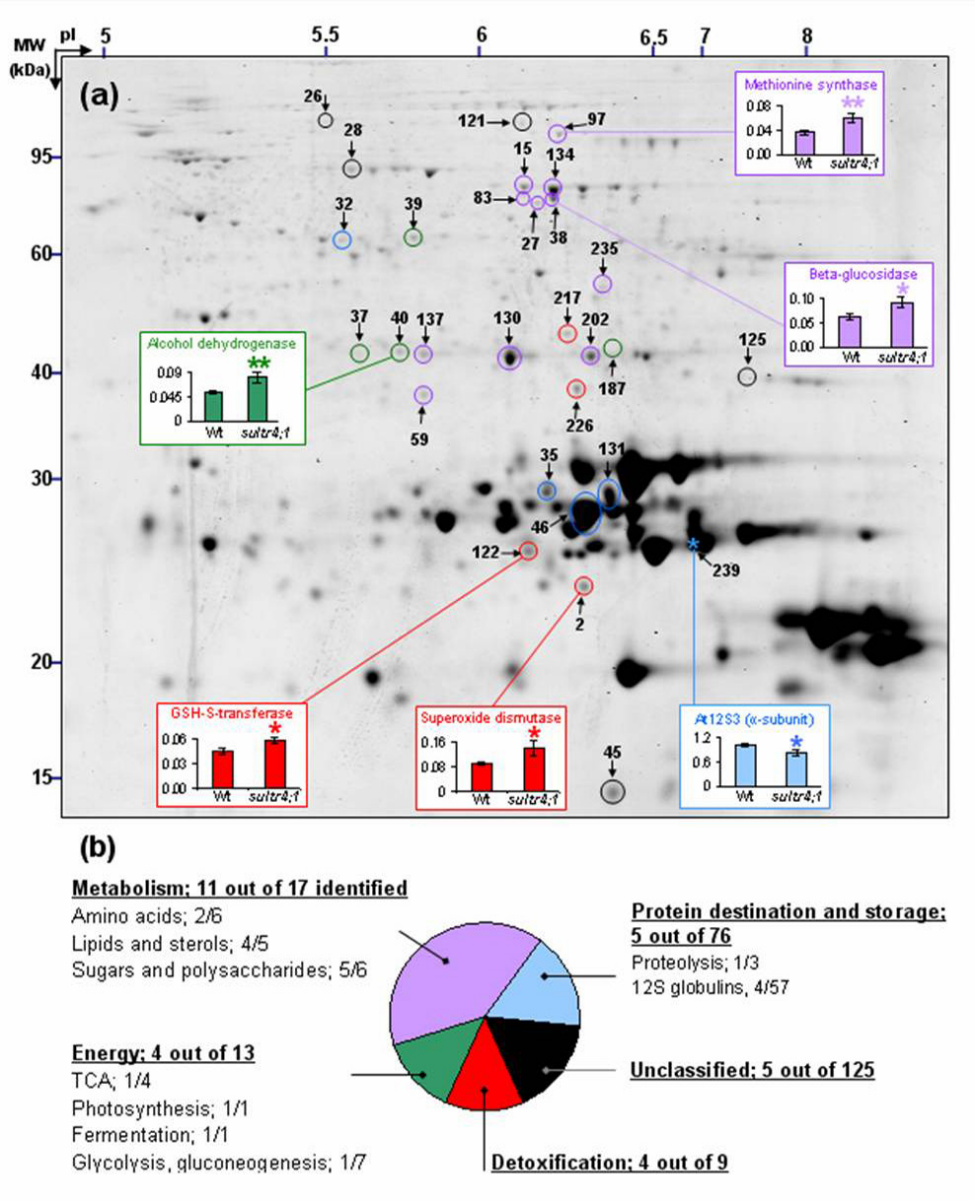
**Proteome variations detected in mature seeds of Sultr4;1**. a) A representative proteome map from mature Arabidopsis seeds is shown. Circles and stars represent proteins whose level respectively increased and decreased in s*ultr4;1 *mutant seeds compared to wild-type. The spots #11, 27, 32, 40, 45, 83, 122, 134, 137, 187 and 226 were identified by using the proteome reference maps from Gallardo et al. [25,26] and Rajjou et al. [27]. The other proteins were identified by mass spectrometry in the present study (see Additional file 7). Colors indicate the functional category of proteins according to the ontological classification of Bevan et al. [29] (see Figure 4b, Additional files 5 and 6). Example of abundance variations for spots 2, 38, 40, 97, 122 and 239 in wild-type and *sultr4;1 *mutant are shown. Each bar represents the mean ± SE of at least three biological replicates. * and ** indicate p < 0.05 and p < 0.01 respectively (variance analysis). b) Distribution of proteins whose abundance varied significantly in each functional category. Within each class, the number of proteins whose abundance vary *versus *the total number of proteins identified is indicated. See Additional file 4 for a complete annotation of the proteome reference map.

To determine if the increased sulphate content in s*ultr4;1 *seeds, putatively due to a reduced vacuolar efflux capability, has influenced seed composition, seed nitrogen and carbon contents were analysed along with the seed proteome complement. In mature s*ultr4;1 *seeds, nitrogen and carbon amounts were unchanged (Figure 2b) but important proteome changes were observed as compared to wild-type. With regard to the proteome analysis, total proteins were extracted from three biological replicates and separated by 2D electrophoresis. Image analysis of Coomassie Blue gels using the Samespots software allowed the quantification of protein abundance for 273 well-resolved spots (see Additional files 4 and 5). Statistical analyses (ANOVA and Levene tests) highlighted 29 spots for which the abundance in s*ultr4;1 *seeds varied significantly compared to wild-type (see Figure 4a and Additional file 6). It is worth noting that among these 29 spots, 28 showed a higher relative abundance level in s*ultr4;1 *seeds, whereas only one spot (spot 239) showed a decreased accumulation.

**Figure 5:**
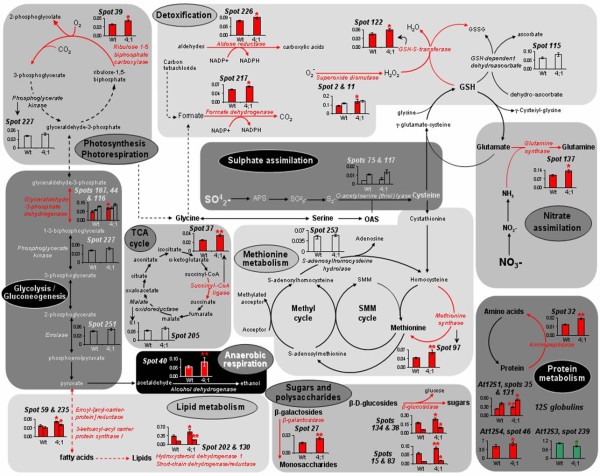
**A summary of metabolic pathways modulated in Sultr4;1 mutant seeds**. Proteins were located in their metabolic pathways according to their annotation http://www.arabidopsis.org, ontological classification [29] and to established features of metabolism. The spots #11, 27, 32, 40, 45, 83, 122, 134, 137, 187 and 226 were identified by using the proteome reference maps from Gallardo et al. [25,26] and Rajjou et al. [27]. The other proteins were identified by mass spectrometry in the present study (see Additional file 7). Bars represent the means of spot level variations ± SE in mature seeds of wild-type and *sultr4;1 *mutant. Significant variations are indicated by colored red (increased levels in *sultr4;1 *seeds) or green (decreased levels) bars. * and ** indicate p < 0.05 and p < 0.01 respectively (variance analysis). GSH, glutathione; GSSG, glutathione disulphide, OAS, O-acetylserine; SMM, *S-*methylmethionine; TCA, tricarboxylic acid cycle.

To study the putative function of the proteins whose abundance varied or remained unchanged in s*ultr4;1 *seeds, we annotated 152 spots detected in 2D gels by using proteome reference maps previously established for Arabidopsis seeds [[25,26] and [27]]. The identity of 59 spots was confirmed by tryptic peptide mass fingerprinting or LC-MS/MS (see Additional file 7). Of the 29 spots varying in the s*ultr4;1 *seed proteome, 25 were identified. The metabolic pathways modulated in mutant seeds compared to wild-type were revealed by classifying the proteins into functional categories according to the gene ontology of Bevan et al. [28] (see Additional files 5 and 6). The distribution into each functional category of proteins whose abundance varied significantly, and the modulated metabolic pathways, are shown in Figures 4 and 5 respectively. In mutant seeds, most of the spots whose abundance varied as compared to wild-type were related to metabolism (eleven spots of 17 identified), detoxification (four spots of nine identified) and energy (four spots of 13 identified).

Within the metabolism category, lipid and sterol metabolism was particularly affected in mutant seeds: among the five spots identified as proteins related to lipid and sterol metabolism, four (spots 59, 235, 202 and 130) showed an increased abundance in mutant seeds. Sugar and polysaccharide metabolism may also be modulated in mutant seeds, since several spots corresponding to enzymes involved in polysaccharide catabolism, a β-glucosidase [spots 15, 38 (Figure 4), 83 and 134] and a β-galactosidase (spot 27), increased in mutant seeds. Finally two proteins of amino acid metabolism were also over-accumulated in the s*ultr4;1 *mutant seeds that correspond to glutamine synthase (spot 137) and methionine synthase (spot 97 in Figure 4).

Another interesting result was the over-accumulation in mutant seeds of several proteins related to stress response mechanisms. Four spots corresponding to enzymes involved in detoxification processes, a glutathione S-transferase (spot 122), an aldose reductase (spot 226), a formate dehydrogenase (spot 212), and a superoxide dismutase (spot 2, Figure 4), were over-accumulated in the *Sultr4;1 *mutant seeds. Moreover, spots corresponding to proteins usually up-regulated during stress response and notably oxidative stress [29-31], such as glyceraldehyde-3-phosphate dehydrogenase (spot 187) and alcohol dehydrogenase (Spot 40 in Figure 4), were increased in abundance in mutant seeds. These results prompted us to estimate the level of oxidation of the glutathione pool in seeds, which is indicative of the redox status. The amounts of reduced glutathione (GSH) and glutathione disulphide (GSSG, oxidized form) were measured in dry mature seeds freshly collected from wild-type and *sultr4;1 *mutant plants (see Additional file 8). The mutant seeds possessed higher GSSG levels (46% against 42% for wild-type seeds), although the increases were not statistically significant.

In contrast to the substantial variations of proteins involved in metabolism and stress-related pathways, there were few changes in storage protein amounts. Of the 57 spots annotated as 12S globulin isoforms, which are the most abundant proteins found in Arabidopsis seeds (i.e. they represent about 80% of total seed protein content), only four varied in abundance between wild-type and mutant seeds. Of these variable spots, three (spots 35 and 131, isoform At12S1 and spot 46, isoform At12S4) and one (spot 239, isoform At12S3, Figure 4) showed respectively an increased and a decreased accumulation in mutant seeds. These variations had no influence on seed protein content since values obtained by the Bradford assay were similar for s*ultr4;1 *and wild-type seeds.

## Discussion

In Arabidopsis, the sulphate transporter group 4 is composed of two members (SULTR4;1 and SULTR4;2), localized in the vacuolar membrane and reported to be expressed in roots, where a role was proposed in the efflux of sulphate from the vacuole [21]. In the present study, we observed that during the reproductive growth phase, *Sultr4;1 *was preferentially expressed in developing Arabidopsis seeds at the transition between embryogenesis and seed filling and presented a relative transcript abundance much higher than *Sultr4;2*, which was expressed at similar levels throughout seed development (see Figure 1 and Additional file 1). These data, along with previous data reporting that SULTR4;1 plays a major role and SULTR4;2 only a small contribution in remobilising the sulphate reserves [21], prompted us to study the role of SULTR4;1 in determining seed composition. We found a remarkably high level of sulphate constrasting with an unchanged sulphur content in mature seeds of a *sultr4;1 *T-DNA mutant (Figure 2), which for the first time implies a function for this transporter in developing seeds. The unchanged seed sulphur content indicates that the increased sulphate level is not related to an enhanced sulphur supply to the developing seed, but rather to a reduction of sulphate utilization/assimilation within the seed. Despite the unchanged sulphur content in mature seeds, we cannot exclude that the composition of sulphur or the amount of sulphate and other sulphur metabolites transported to developing seeds could be different between the wild-type and the *sultr4;1 *mutant. However, as no significant difference in seed yield, leaf surface or onset of flowering were observed, the increased sulphate/sulphur ratio in *sultr4;1 *seeds appears not to be related to a drastic perturbation of *sultr4;1 *vegetative growth. The increased sulphate/sulphur ratio in *sultr4;1 *seeds is possibly the consequence of a reduced remobilization (i.e. assimilation) of the vacuolar sulphate pool within the developing seed. The vacuolar localisation of the transporter in seeds has not yet been proven. We therefore cannot exclude that the protein could be localised in different compartments in seeds. However, the increased sulphate/sulphur ratio in mutant mature seeds is consistent with previous data showing that, in roots of *sultr4;1 *mutants, sulphate is retained as a consequence of defects in sulphate efflux from the vacuole [21]. It should also be noted, that other genes encoding sulphate transporters are expressed in developing seeds [22]. In particular, the *Sultr3;4 *gene encoding a putative plasma-membrane sulphate transporter is strongly expressed during early seed filling at similar stages to those of S*ultr4;1*. Interestingly, the seed proteome profile of the *sultr4;1 *mutant differs from that of a s*ultr3;4 *mutant (unpublished data), indicating that SULTR4;1 and SULTR3;4 may have distinct roles/subcellular localization during seed filling.

Overall nitrogen and protein levels were unaltered in *sultr4;1 *seeds, but a dissection of the *sultr4;1 *seed proteome revealed that ~10% of the minor protein spots detected in 2D gels varied in abundance (see Additional files 5 and 6), thus reflecting metabolic modifications (Figure 5). In particular, several proteins with roles in the oxidative stress response were up-accumulated. An oxidative stress response has frequently been observed during sulphate deprivation [32,33], notably in sulphur-deficient seeds [34]. In the present study, most of the proteins with roles in the oxidative stress response are involved in detoxification mechanisms (Figure 5). Among them were a scavenger of free oxygen radicals (superoxide dismutase, spot 2 in Figure 4), and the glutathione *S*-transferase isoform 6 (spot 122 in Figure 4) that may play a protective role against oxidative damage due to hydrogen peroxide [35]. This may represent an adaptive response to oxidative stress in s*ultr4;1 *seeds as these enzymes detoxify some of the toxic compounds, such as reactive oxygen species (ROS), produced by oxidative stress.

At low levels, ROS participate in cellular signaling. In Arabidopsis seeds, abundant proteins were identified that act as scavengers of ROS generated during seed development, thereby counteracting their deleterious effects [36]. However, if ROS accumulate to harmful levels, seeds lose their ability to control ROS and cannot restart their metabolism [37], probably because ROS cause irreversible oxidative damage to lipids, proteins, and DNA. We have observed that *Sultr4;1 *seeds lose their viability during storage much more rapidly than wild-type seeds. Indeed, seeds collected from homozygous *Sultr4;1 *plants did not germinate after two years of storage at room temperature, while almost all seeds collected at the same time from wild-type plants germinated. A tetrazolium test confimed the loss of viability of the *Sultr4;1 *mutant seeds (see Additional file 9), which could be linked to their increased sensitivity to oxidative stress during storage, when ROS are known to be continuously produced [38]. However, the up-regulation of enzymes involved in ROS removal that we see in s*ultr4;1 *seeds probably reflects seed developmental events and not changes during sample storage, as the seeds subjected to proteomics were frozen in liquid nitrogen immediately after harvest.

In summary, our data suggest sulphate remobilization from the vacuole to the other cell compartments is important for the seed's defence against abiotic oxidative stress during seed development and storage. This could also apply to other tissues, since in Arabidopsis roots and shoots, cadmium stress induces an up-regulation of *Sultr4;1 *gene expression concomitantly with an oxidative stress response [39]. Also, in *Brassica juncea *seedlings, *Sultr4;1 *gene expression was up-regulated in response to arsenic-induced stress [40,41], which leads to the generation of ROS through the conversion of arsenate to arsenite [42].

Besides proteins related to the oxidative stress response, an up-accumulation of proteins involved in the biosynthesis of fatty acids and lipids was revealed. They correspond to enoyl- [acyl-carrier-protein] reductase (spot 59 in Figure 4), two isoforms of hydroxysteroid dehydrogenase 1 (HSD1, spots 130 and 202), and ketoacyl carrier protein synthase I (spot 235). An up-regulation of a ketoacyl carrier protein synthase was also observed in developing Arabidopsis seeds under sulphur-starved conditions [34]. It is well known that under stress conditions, toxic oxygen derivatives are produced that inactivate enzymes and damage important cellular components, such as membranes *via *lipid peroxidation and fatty-acid de-esterification. In this context, the up-regulation of enzymes involved in fatty acid and lipid biosynthesis may represent a mechanism to repair stress-induced membrane damage. In support of this, Li et al. [43] showed that transgenic Arabidopsis lines overexpressing HSD1 have an increased tolerance to salt stress.

Interestingly, an up-regulation in proteins of the energy and carbon metabolism was observed in s*ultr4;1 *seeds. These are involved in glycolysis (glyceraldehyde-3-phosphate dehydrogenase, spot 187), photosynthesis or photorespiration (ribulose 1-5 biphosphate carboxylase large chain, spot 39), and anaerobic fermentation (alcohol dehydrogenase, spot 40 in Figure 4). Since an up-accumulation of enzymes involved in fatty acid and lipid biosynthesis was also observed in *sultr4;1 *seeds, it is possible that energy production and glycolysis were enhanced in *sultr4;1 *seeds to provide carbon skeletons for the synthesis of fatty acid precursors in these seeds [44]. Furthermore, members of the glycosyl hydrolases that specifically release sugar from oligosaccharides [β-glucosidase, spots 15, 38 (Figure 4), 83, 134; and β-galactosidase, spot 27 (Figure 5)] were up-accumulated in *sultr4;1*, probably as a way to sustain glycolysis and fatty acid biosynthesis.

This study demonstrated that the accumulation of storage proteins occurs in s*ultr4;1 *mutant seeds (see Additional files 5). In 2D-gels, these proteins are located in the 20 to 30 kDa range (see Figure 4a and Additional files 4 and 5). Only a few storage proteins differ in abundance from wild-type seeds and these correspond to globulin 12S isoforms (Figure 5). The only protein whose level decreased was the At12S3 isoform (spot 239 in Figure 4a) and the three proteins whose level increased were At12S1 (spots 35 and 131) and At12S4 (spot 46). Interestingly, the isoforms whose level increased have a relatively poor sulphur amino acid content (2.4 and 2.8%, respectively) compared to At12S3 (4.3% of total amino acid residues) whose level decreased. A similar increase in sulphur-poor seed proteins was observed during sulphur deprivation [34,45-47]. This indicates that, in sulphur-sufficient conditions, sulphate exported from the vacuole *via *SULTR4;1 participates but is not limiting for storage protein synthesis. A possibility to be considered is that SULTR4;2 could also participate to vacuolar sulphate release for storage protein synthesis. However, expression patterns of the gene and data from the literature suggest SULTR4;2 may have only a supplementary role in sulphate export from the vacuole [21].

Interestingly, an increased accumulation of a methionine synthase (spot 97, Figure 4) was observed in s*ultr4;1 *seeds. It is also worth noting that under sulphate-deprived conditions, despite the almost complete depletion of cysteine, the plant is able to keep methionine levels constant [32,48,49]. This demonstrates the plant's capacity to regenerate methionine through alternative pathways under sulphate-deficient conditions and upon cysteine restriction. Methionine can be regenerated through methylation of homocysteine produced by transmethylation *via *a reaction catalyzed by S-adenosylhomocysteine hydrolase (Figure 5). Moreover, the use of the 4-carbon moiety of *S*-adenosylmethionine for the synthesis of polyamines and ethylene also is accompanied by recycling of the methylthio moiety and regeneration of methionine [50]. Interestingly, the protein spot identified in the present study corresponds to a cytosolic isoform involved in the regeneration of methionine from homocysteine produced in the course of the activated methyl cycle [51]. In *sultr4;1 *mutant seeds, the up-regulation of methionine synthesis could be a way to sustain seed protein synthesis. Moreover, maintaining the synthesis of proteins rich in methionine may be particularly important in stress conditions, since protein-endogenous methionine reacts with ROS, as a scavenger, to form sulphoxide (MetSO) without loss of activity of the corresponding protein [52,53].

## Conclusions

This study revealed a role for SULTR4;1 in determining sulphate content of mature Arabidopsis seeds, possibly because it controls the efflux of sulphate from the vacuoles for metabolism in other cell compartments [21]. Specific seed metabolic features were also revealed through a fine dissection of the seed proteome of a *sultr4;1 *mutant (Figures 4 and 5). In particular, an up-regulation of proteins involved in the oxidative stress response was revealed that suggests a function of SULTR4;1 in maintaining redox homeostasis during seed development. The maintenance of redox homeostasis is of crucial importance in plant tissues, and even more in seeds that have the ability to tolerate dehydration in the course of their development. This remarkable ability allows the orthodox seed, i.e., seed that can be stored in a state of low moisture content [54], to survive for several years in a desiccated state with metabolic activities at a standstill, until the environmental conditions become optimal for its germination. As seed desiccation enhances ROS formation, mechanisms that protect from oxidative stress are therefore crucial for maintaining cellular integrity and homeostasis [55]. Glutathione, which is a sulphur-derived product, is a major plant antioxidant. During desiccation, it is converted into the oxidized form glutathione disulphide (GSSG) and the balance between these two compounds is crucial for homeostasis and seed survival. Indeed, the GSSG *versus *GSH ratio can be applied to assess seed viability, since an increased ratio was typically correlated with viability loss in *Pisum sativum *seeds [54]. In the present study, we observed that mutant seeds, freshly collected at the dry mature stage, possessed higher GSSG levels, although the increases were not statistically significant (see Additional file 8). It is worth noting that generally, the level of GSSG is much higher in mature seeds than in other plant parts. For example, only 10% of total glutathione is oxidized in Arabidopsis seedlings [56,57]. This indicates that the glutathione pool is relatively highly oxidized in mature Arabidopsis seeds compared to other plant organs. Despite the absence of significant changes in GSH and GSSG levels between wild-type and mutant seeds, we cannot exclude a localized disturbance of the redox balance in particular tissue or subcellular compartments of *sultr4;1 *mutant seeds. Indeed, as reported by Meyer et al. [58], measurements of GSSG/GSH ratios in plant tissues are prone to artefacts since they do not take into consideration the subcellular compartmentalization. Also, partial oxidation of glutathione during extraction is difficult to avoid and a slight increase in GSSG concentration, whilst barely detectable, would suffice to shift the glutathione redox potential to significantly less reducing values [59]. For these reasons, and because GSSG/GSH ratio in mature seeds is not representative of the events occurring at specific stages of seed development or during storage, future studies will be necessary to evaluate the redox status in different cell types throughout seed development and storage. Very little is known about the exact role of sulphate released from the seed vacuoles in the course of seed development in maintaining cellular homeostasis during seed development. Although further studies will be necessary, this paper is the first contribution on this topic.

## Methods

### Plant materials and growth conditions

We studied Arabidopsis T-DNA insertion lines (Columbia ecotype, Col-0) from the Nottingham Arabidopsis Stock Center (Nottingham University, UK) for the gene *4;1 *(AT5G13550). The T-DNA line has the following accession number: SALK-120920. Further information about mutant accession and T-DNA localization can be found at http://arabidopsis.info and http://atidb.org. The T-DNA insertion line used in this study was not back-crossed and a single allele was used, but conscious of the presence of other insertions in the genome and of the limitations of using only one allele, we have taken care to compare several mutant plants to several wild-type plants derived from plants segregating the mutant allele, to minimize the possible effect of insertions elsewhere in the genome. The plants were grown under sulphur-sufficient conditions (peat-perlite mixture) in a greenhouse with supplemental light to 16 h/day, and fertilized three times a week by subirrigation (N-P-K: 20-20-20). For genotyping, DNA was extracted from a leaf disk by using the CTAB method from Doyle and Doyle [60]. A PCR screen was performed by using specific primers for each gene binding upstream and downstream of the predicted T-DNA insertion as well as one primer binding in the left border region of the corresponding T-DNA. The following primers were used: Lba2-for(5'-GAACAACACTCAACCCTATCTC-3') for the T-DNA, and *4;1*-for(5'-GCA TTCGTTATCCACGAGTCTG-3') and *4;1*-rev(5'-CTCTGTACGTATTGTAGACA CAC-3') for the gene-specific primers.

### Phenotypic characters

Rosette leaf area and seed yield were measured from at least three individual plants. Leaf area was quantified from the rosette scan at flowering using the Visilog 5.4 software (Noesis, Les Ulis, France). The onset of flowering was scored and expressed in °C/day. Seed weight was determined from three seed samples of 10 mg (around 400 seeds) collected on three individual plants. All data were submitted to statistical analyses (variance analysis) using the Statistica 7.0 software (Maisons-Alfort, France). Only differences with *P *values < 0.05 were considered significant.

### Tetrazolium test

Seed viability was estimated by using the tetrazolium test, which differentiates viable from dead tissues of seed embryos on the basis of dehydrogenase activity (respiration enzymes) that reduces tetrazolium salt to formazan in viable tissues. The mutant and wild-type seeds collected at the same time and stored under the same conditions for 25 months were imbibed 24 hours in water. Twenty five embryos from wild-type and mutant seeds were isolated and placed for one hour in a 0.25% tetrazolium salt solution (2,3,5-triphenyltetrazolium chloride).

### RNA extraction and quantitative real-time PCR

Silique and seed samples at three stages of development (pods at 7 and 10 days after flowering, and mature seeds) and tissue samples (flowers, roots, leaves, stems) were collected on two independent batches of plants. Frozen tissues were ground in liquid nitrogen and total RNA was extracted using the method described by Chang *et al*. [61]. RNAs (10 μg) were incubated in presence of 10 units of RNAse-free RQ1 DNAse (Promega, Madison, USA). Non reverse-transcribed RNA samples were checked for absence of contaminating genomic DNA by PCR using primers for the constitutively expressed elongation factor alpha-chain gene (At5g60390). DNA-free RNA was converted into first-strand cDNA. Samples were reverse-transcribed using the iScript cDNA synthesis Kit (Bio-Rad, Hercules, USA). The quantitative RT-PCR was performed using the real-time SYBR Green method on a BioRad iQ5 thermal cycler, using iQ SYBR Green Supermix (BioRad) and the following specific primers: *4;1*-for(5'-CACTTGACAATAGCAAGATCAGG-3'), *4;1*-rev(5'-CTCTGTACGT ATTGTAGACACAC-3'), 4;2-for(5'-CTAGCAAGAGCAGGCATTGTGGA-3') and 4;2-rev(5'-CTTGGACTGCGTCATGTACTCTC-3'). To establish the presence of a single PCR product and the absence of primer-dimers, melting curve analysis (i.e. heat dissociation of oligonucleotides) was applied immediately after PCR by heating PCR products from 59°C to 96°C. Normalization for cDNA quantity was performed for each template with an Elongation factor gene (At5g60390) as control gene using the relative standard curve method (delta CT) according to the Biorad instructions. The expression stability of the control gene in the different test samples was verified by comparison with two other constitutively expressed genes encoding an ubiquitin (At4g27960) and a protein phosphatase (At1g13320) (data not shown). The following control primers were used: for(5'-GATTGCCACACCTCTCACATTGCAG-3') and rev(5'-GCTCCTTCTCAATCTCCTT ACCAG-3') for the *elongation factor *gene, for(5'-CCAAGGTGCTGCTATCGATCTGT-3') and rev (5'-AGGTCCGAGCAGTG GACTCG-3') for the *ubiquitin *gene and for(5'-ATCGCTTCTCGCTCCAGTAATG-3') and rev(5'-GACTATCGGAATGAGAGATTGC-3') for the *protein phosphate *gene. This method was also applied to assess transcript level changes in mature seed of *sultr4;1 *mutant compared to wild-type. A 94-99% decrease was observed in *sultr4;1 *seeds.

### Determination of anion and N C S contents in mature seeds

Mature seeds harvested from three individual plants were ground (50 mg) in liquid nitrogen. Anions were extracted in Milli-Q water heated at 70°C for 20 min. The seed extract was centrifugated at least 3 times at 20,000 g for 10 min at 4°C. Anion content of the final clear supernatant was determined by high performance ionic chromatography (LC20 Dionex) using a IonPaq AS11 column and a sodium hydroxide linear gradient (1 to 22 mM), as described [17]. Contents of total nitrogen, carbon and sulphur in the dried powder of seed samples were measured were measured using an elemental analyzer (Vario EL; Elemental analyser system). All data were submitted to statistical analyses (variance analysis) using the Statistica 7.0 software (Maisons-Alfort, France). Only differences with *P *values < 0.05 were considered significant.

### Glutathione content and redox state

20 mg of seeds were used for isolation of total or oxidized thiols as described in Fey et al. [57]. Derivatization of thiols with monobromobimane (Calbiochem) and separation of thiol derivates were performed according to Wirtz et al. [62] using the same HPLC-system. The Empower™ software (Waters) was used for data acquisition and processing.

### Total protein extraction and 2D electrophoresis

The seed samples (dry mature stage) subjected to proteomics were immediately frozen in liquid nitrogen after harvest to avoid changes in protein abundance that can be induced by storage. Total proteins were extracted as described by Gallardo *et al.*, [63], from 20 mg of mature seeds collected on four individual plants. Protein concentration was measured according to Bradford [64]. Eight gels (four biological replicates and two technical replicates from each biological replicates) were performed for each wild-type and mutant lines. A constant volume (57 μl) of the protein extracts (around 200 μg of proteins) was used for isoelectrofocusing, that corresponded to a constant seed weight (2 mg). Proteins were separated in duplicates using gel strips forming an immobilized non-linear pH 3 to 10 gradient (Immobiline DryStrip, 24 cm; GE Healthcare/Amersham Biosciences). Strips were rehydrated in the IPGphor system (GE Healthcare/Amersham Biosciences) for 7 h at 20°C with the thiourea/urea lysis buffer containing 2% (v/v) Triton X-100, 20 mM DTT and the protein extracts. IEF was performed at 20°C in the IPGphor system for 7 h at 50 V, 1 h at 300 V, 2 h at 3.5 kV and 7 h at 8 kV. Prior to the second dimension, each gel strip was incubated at room temperature for 2 × 15 min in 2 × 15 ml equilibration buffer as previously described [26]. Proteins were separated in vertical polyacrylamide gels according to [26].

### Protein staining and quantification

Gels were stained with Coomassie Brilliant Blue G-250 (Bio-Rad, Hercules, CA, USA) according to Mathesius et al. [65] Image acquisition was done using the Odyssey Infrared Imaging System (LI-COR Biosciences, Lincoln, NE) at 700 nm with a resolution of 169 μm. Image analyses and spot volume quantification were carried out with the Progenesis SameSpots version 2.0 software (Nonlinear Dynamics, Newcastle upon Tyne, UK) according to the instruction manual. For each gel, normalized spot volumes were calculated as the ratio of each spot volume to the total spot volume of the gel (arbitrary unit). Eight gels (four biological replicates and two technical replicates from each biological replicates) were analysed for each wild-type and mutant lines. Molecular masses (Mr) and isoelectric points (p*I*) were calculated according to the migration of standard proteins (Bio-Rad, Bio-Rad, Hercules, USA). All data were submitted to statistical analyses (variance analysis and Levene's test) using the Statistica 7.0 software (Maisons-Alfort, France). Only differences with p-values < 0.05 were considered significant.

### Protein identification

Spots were annotated using proteome reference maps previously established for Arabidopsis mature seeds ([25-27], http://www.seed-proteome.com). In the present work, we confirmed identification of 53 spots by nano-LC-MS/MS (Q-TOF-Ultima Global equipped with a nano-ESI source coupled with a Cap LC nanoHPLC, Waters Micromass, Waters, Saint Quentin en Yvelines, France) as described by Gallardo et al. [66]. Detailed information about protein digestion, mass spectrometry data acquisition are in Additional file 7. Peak lists of precursor and fragment ions were matched to proteins in the NCBI non redundant database (March 2008, 7,387,702 sequences; 2,551,671,261 residues, taxonomy of Arabidopsis) using the MASCOT version 2.2 program (Matrix Science, London). The MASCOT search parameters are described in Additional file 7. Only matches with individual ion scores above 20 were considered.

## Competing interests

The authors declare that they have no competing interests.

## Authors' contributions

HZ participated in experimental design, conducted the bulk of the experimental work, performed the qRT-PCR experiment, 2D-electrophoresis analyses, phenotypic T-DNA mutant characterisation and statistical analyses and drafted the manuscript. JCD performed the HPIC analyses and was involved in revising the manuscript critically. MW and RH performed CNS and GSSG/GSH analyses and were involved in revising the manuscript critically. RT participated in designing and surpervising the study and helped to draft the manuscript. KG conceived, designed and supervised the study and participated in drafting the manuscript. All authors have read and approved the final manuscript.

## Supplementary Material

Additional file 1 **Comparison of the qRT-PCR data obtained in the present study for the Arabidopsis genes belonging to the group 4 of sulphate transporters with the corresponding data from a publicly available expression atlas **[22]. A drawing was made from a photo of the Arabidopsis plant and, for each gene, gene expression in roots, rosette and cauline leaves, entire flower, stem, and seeds, from the publicly available expression atlas of Arabidopsis was mapped as colors. The qRT-PCR expression data are indicated by colored squares. These expression data were normalized to the highest expression value set to 1. A color scale represents variations in transcript abundance for each gene, in which red represents the highest expression and white the lowest expression. Missing values are in grey. Microarray data showing *Sultr4;1 *expression in roots of young seedlings under sulfur-starvation conditions, *versus *normal conditions (AthXpressionist@CSB.DB, http://csbdb.mpimp-golm.mpg.de/csbdb/dbxp/ath/ath_xpmgq.html) were also included. Click here for file

Additional file 2 **Structure of the *Sultr4;1 *gene, T-DNA insertion site and trancript level (qRT-PCR) in wild-type and mutant lines**. a) Structure of *Sultr4;1 *gene is shown with the insertion site of the T-DNA (SALK-120920). Exons are indicated by white boxes, untranslated regions by dark boxes and T-DNA insertion by a dashed box. b) The relative mRNA quantity was estimated by qRT-PCR in mature seeds of wild-type (Wt) and *sultr4;1 *mutant plants. Bars represent the mean ± SE of three biological replicates. Click here for file

Additional file 3 **Estimation of anion levels in the seed compartment (a) and per 100 seed (b) for wild type (Wt) and the *sultr4;1 *mutant**. a) Anion levels correspond to seed anion content * seed mass per plant. b) Anion levels correspond to seed anion content * 100-seed weight. Seed anion content was determined by high performance ionic chromatography. Results are representative of three biological experiments. Bars represent the mean ± SE of six measurements (at least two technical replicates from each biological replicate). * and ** indicate p < 0.05 and p < 0.01 respectively (variance analysis). Click here for file

Additional file 4 **Proteome map of mature Arabidopsis seeds Col-0, wild-type**. Total soluble proteins were electrofocused in a non-linear pH gradient from 3 to 10, then separated by SDS-PAGE (10%) (see Additional files 5 and 6). Click here for file

Additional file 5 **Proteomics datasets from mature seeds in wild-type and mutant lines**. This table includes: MW and pI of the 273 detected proteins, their accumulation ratios (mutants *versus *wild-type), the ANOVA and Levene P values, their annotation and ontological classification [29]. Click here for file

Additional file 6 **Detailed information concerning proteins whose abundances vary significantly between mutant and wild-type mature seeds**. This table includes: MW and pI of the 29 proteins whose abundance vary significantly, their annotation, ontological classification [29], and their accumulation level in wild-type and mutant seeds. Bars represent the mean ± SE of six measurements (at least two technical replicates from each biological replicate). ANOVA and significant differences are labelled: *, ** and *** indicate p < 0.05, p < 0.01 and p < 0.001 respectively. Click here for file

Additional file 7 **Detailed information about LC-MS/MS protein identification**. This table includes detailed information about protein digestion, mass spectrometry, data acquisition, MASCOT search parameters, peptide sequences and identification scores. Click here for file

Additional file 8 Total glutathione, glutathione disulphide (GSSG), reduced glutathione (GSH) in mature seeds of wild type and *sultr4;1 *mutant lines. Click here for file

Additional file 9 **Viability estimation of dry mature seeds freshly collected from the *sultr4;1 *mutant and wild type plants by using the tetrazolium test**. Pictures are representative of twenty five embryos from wild-type and mutant seeds placed for one hour in a tetrazolium salt solution (2,3,5-triphenyltetrazolium chloride). Red coloration allows visualizing the living part of the embryo. All embryos isolated from wild-type seeds were stained red, thus attesting to their viability. In contrast, all embryos from mutant seeds were unstained, indicating a loss of viability. Click here for file
