# Supplementary figures and images for: Sultr4;1 mutant seeds of Arabidopsis have an enhanced sulphate content and modified proteome suggesting metabolic adaptations to altered sulphate compartmentalization
Source: BMC Plant Biol. 2010 Apr 28;10:78. doi: 10.1186/1471-2229-10-78 (PMC3095352; doi:10.1186/1471-2229-10-78)

Color scale representing variations in transcript abundance for each gene:

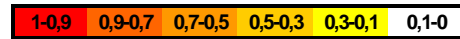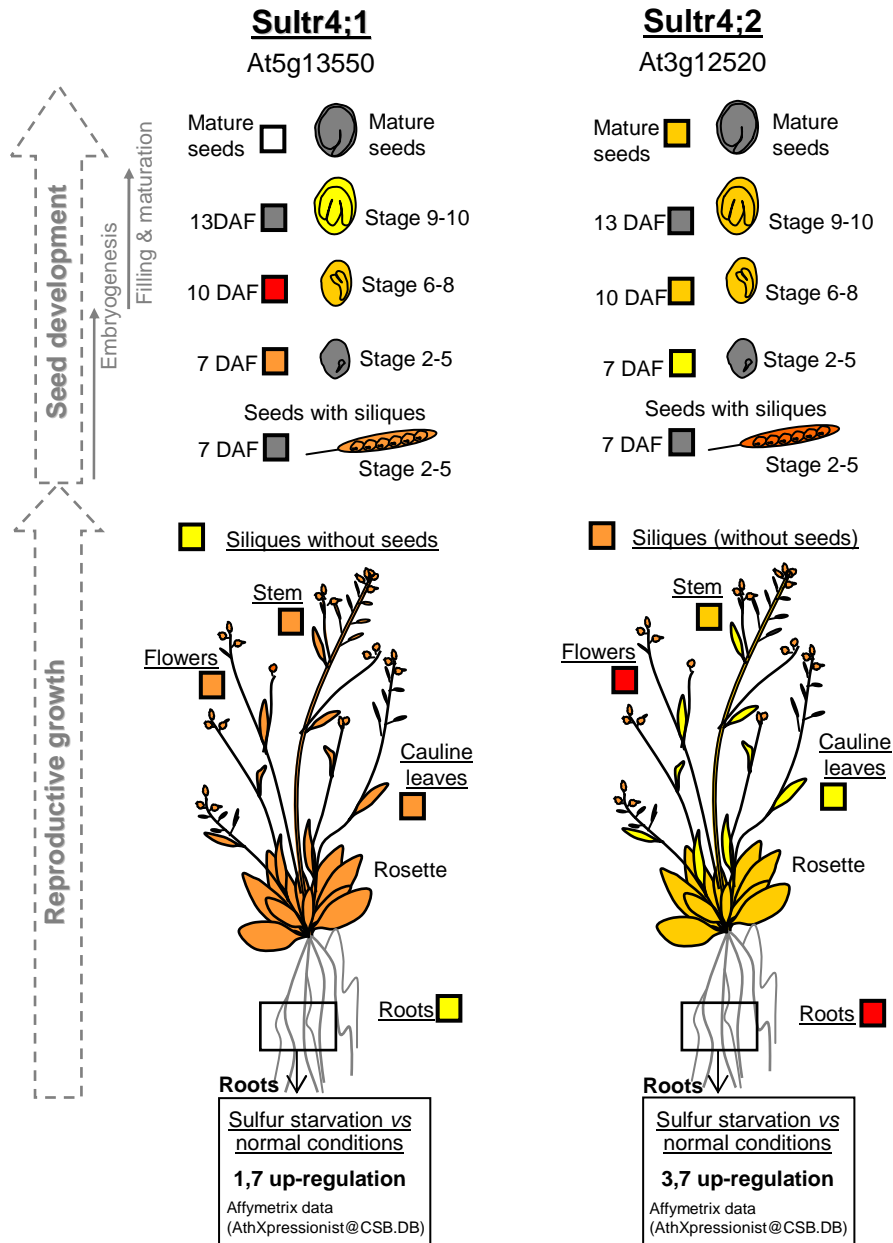

Supplement: Additional file 1 — Comparison of the qRT-PCR data obtained in the present study for the Arabidopsis genes belonging to the group 4 of sulphate transporters with the corresponding data from a publicly available expression atlas [22]. A drawing was made from a photo of the Arabidopsis plant and, for each gene, gene expression in roots, rosette and cauline leaves, entire flower, stem, and seeds, from the publicly available expression atlas of Arabidopsis was mapped as colors. The qRT-PCR expression data are indicated by colored squares. These expression data were normalized to the highest expression value set to 1. A color scale represents variations in transcript abundance for each gene, in which red represents the highest expression and white the lowest expression. Missing values are in grey. Microarray data showing Sultr4;1 expression in roots of young seedlings under sulfur-starvation conditions, versus normal conditions (AthXpressionist@CSB.DB, http://csbdb.mpimp-golm.mpg.de/csbdb/dbxp/ath/ath_xpmgq.html) were also included. [file 1471-2229-10-78-S1.PDF]

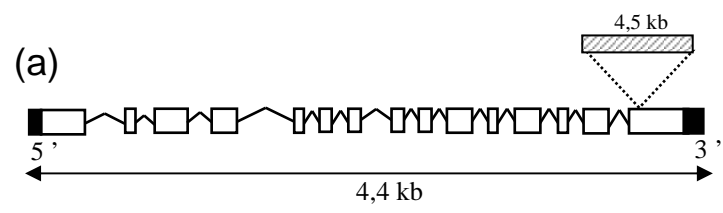

(b) Relative transcript abundance of *Sultr4;1*

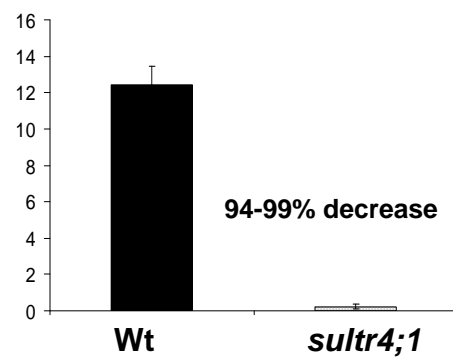

Supplement: Additional file 2 — Structure of the Sultr4;1 gene, T-DNA insertion site and trancript level (qRT-PCR) in wild-type and mutant lines. a) Structure of Sultr4;1 gene is shown with the insertion site of the T-DNA (SALK-120920). Exons are indicated by white boxes, untranslated regions by dark boxes and T-DNA insertion by a dashed box. b) The relative mRNA quantity was estimated by qRT-PCR in mature seeds of wild-type (Wt) and sultr4;1 mutant plants. Bars represent the mean ± SE of three biological replicates. [file 1471-2229-10-78-S2.PDF]

(a) Total seed anion amount ( $\mu\text{mol}$ ) per plant

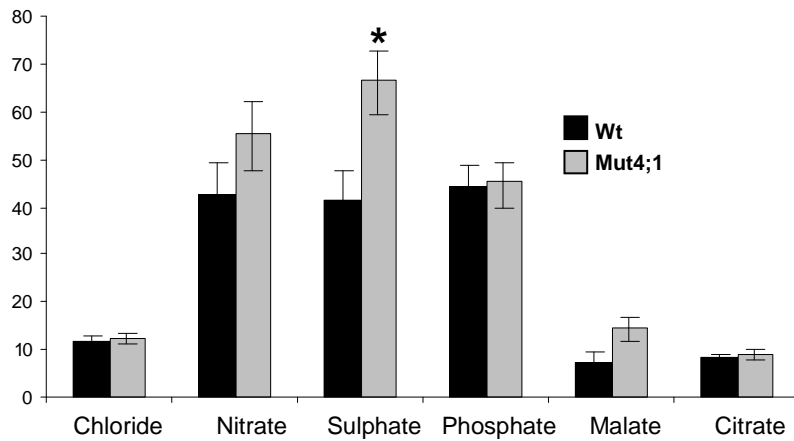

(b) Anion amount ( $\mu\text{mol} \cdot 100\text{-seeds}^{-1}$ )

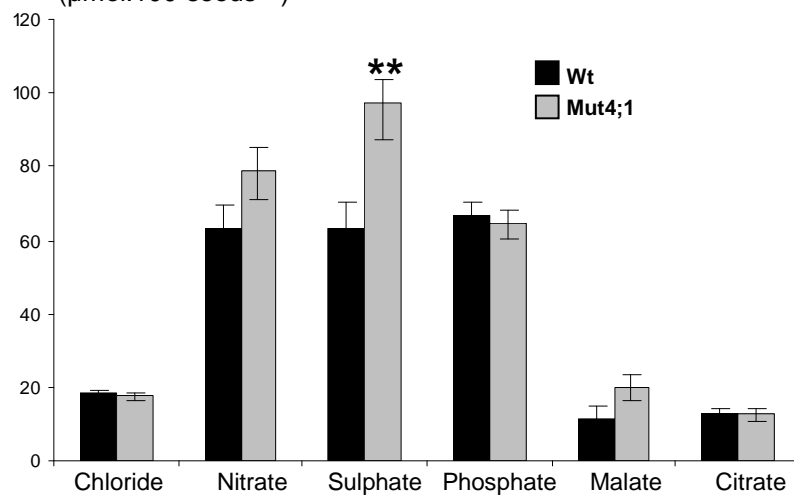

Supplement: Additional file 3 — Estimation of anion levels in the seed compartment (a) and per 100 seed (b) for wild type (Wt) and the sultr4;1 mutant. a) Anion levels correspond to seed anion content * seed mass per plant. b) Anion levels correspond to seed anion content * 100-seed weight. Seed anion content was determined by high performance ionic chromatography. Results are representative of three biological experiments. Bars represent the mean ± SE of six measurements (at least two technical replicates from each biological replicate). * and ** indicate p < 0.05 and p < 0.01 respectively (variance analysis). [file 1471-2229-10-78-S3.PDF]

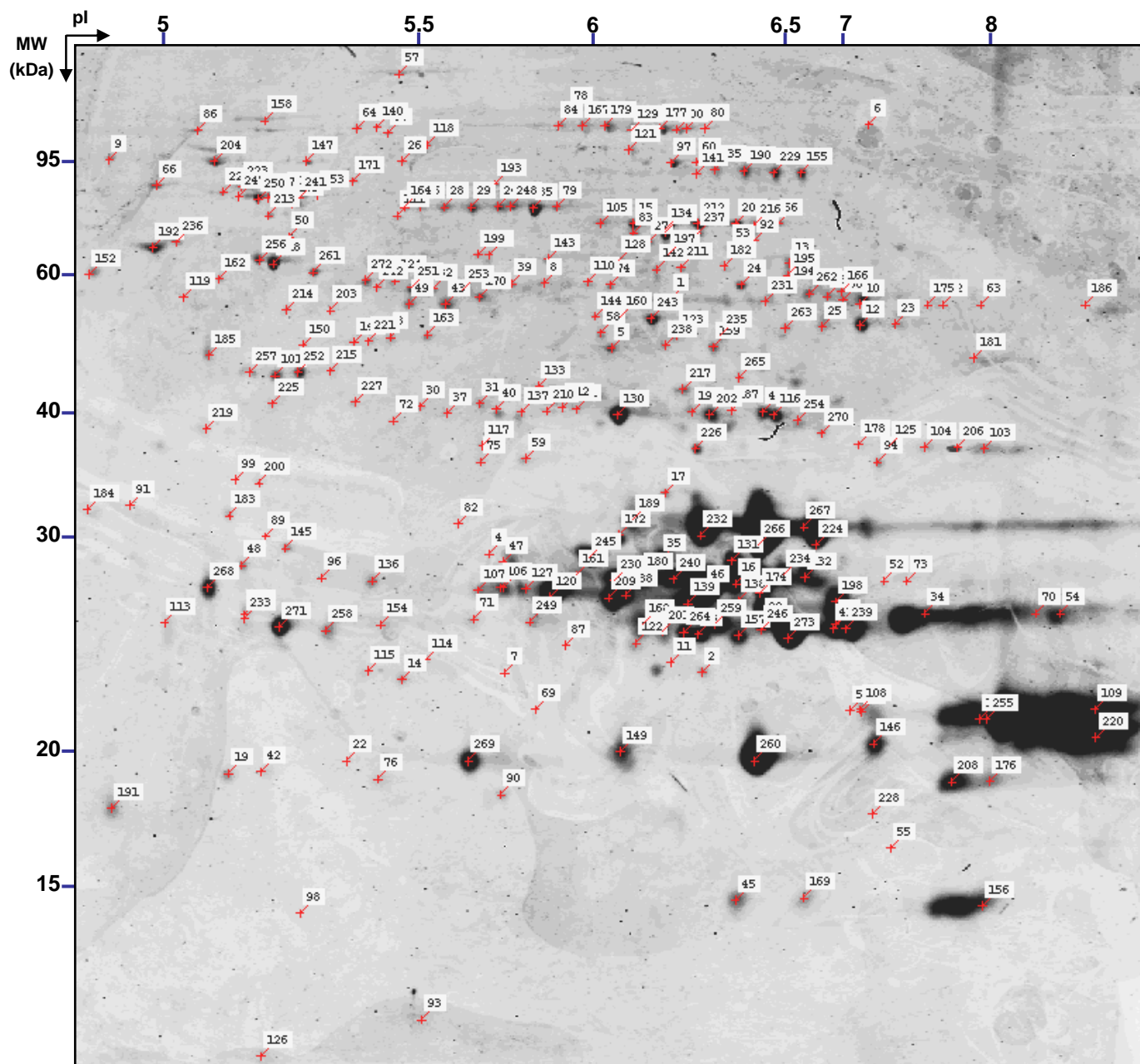

Supplement: Additional file 4 — Proteome map of mature Arabidopsis seeds Col-0, wild-type. Total soluble proteins were electrofocused in a non-linear pH gradient from 3 to 10, then separated by SDS-PAGE (10%) (see Additional files 5 and 6). [file 1471-2229-10-78-S4.PDF]

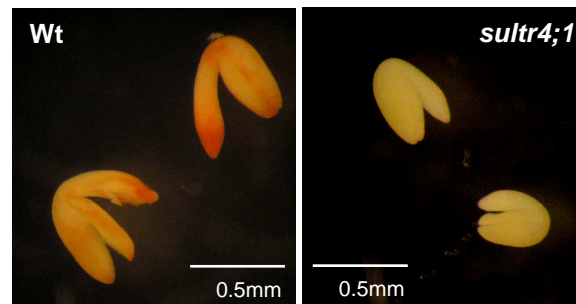

Supplement: Additional file 9 — Viability estimation of dry mature seeds freshly collected from the sultr4;1 mutant and wild type plants by using the tetrazolium test. Pictures are representative of twenty five embryos from wild-type and mutant seeds placed for one hour in a tetrazolium salt solution (2,3,5-triphenyltetrazolium chloride). Red coloration allows visualizing the living part of the embryo. All embryos isolated from wild-type seeds were stained red, thus attesting to their viability. In contrast, all embryos from mutant seeds were unstained, indicating a loss of viability. [file 1471-2229-10-78-S9.PDF]
